# Sevoflurane-Induced Apoptosis in the Mouse Cerebral Cortex Follows Similar Characteristics of Physiological Apoptosis

**DOI:** 10.3389/fnmol.2022.873658

**Published:** 2022-04-08

**Authors:** Qi Wang, Yuan Li, Hong Tan, Yingwei Wang

**Affiliations:** Department of Anesthesiology, Huashan Hospital, Fudan University, Shanghai, China

**Keywords:** sevoflurane, neonates, neuronal apoptosis, Akt, FoxO1, PUMA

## Abstract

General anesthetics are capable of inducing neuronal apoptosis during the rapid synaptogenesis of immature mammalian brains. In this vulnerable time window, physiological apoptosis also occurs to eliminate excess and inappropriately integrated neurons. We previously showed that physiological and ketamine-induced apoptosis in mouse primary somatosensory cortex (S1) followed similar developmental patterns. However, since sevoflurane is more widely used in pediatric anesthesia, and targets mainly on different receptors, as compared with ketamine, it is important to determine whether sevoflurane-induced apoptosis also follows similar developmental patterns as physiological apoptosis or not. Mice at postnatal days 5 (P5) and P9 were anesthetized with 1.5% sevoflurane for 4 h, and the apoptotic neurons in S1 were quantitated by immunohistochemistry. The results showed that sevoflurane raised the levels of apoptosis in S1 without interfering with the developmental patterns of physiological apoptosis. The cells more vulnerable to both physiological and sevoflurane-induced apoptosis shifted from layer V pyramidal neurons at P5 to layers II–IV GABAergic neurons by P9. The magnitude of both sevoflurane-induced and physiological apoptosis was more attenuated at P9 than P5. To determine whether the Akt-FoxO1-PUMA pathway contributes to the developmental decrease in magnitude of both physiological and sevoflurane-induced apoptosis, Western blot was used to measure the levels of related proteins in S1 of P5 and P9 mice. We observed higher levels of antiapoptotic phosphorylated Akt (p-Akt) and phosphorylated FoxO1 (p-FoxO1), and lower levels of the downstream proapoptotic factor PUMA in control and anesthetized mice at P9 than P5. In addition, the Akt-FoxO1-PUMA pathway may also be responsible for sevoflurane-induced apoptosis. Together, these results suggest that magnitude, lamination pattern and cell-type specificity to sevoflurane-induced apoptosis are age-dependent and follow physiological apoptosis pattern. Moreover, The Akt-FoxO1-PUMA pathway may mediate the developmental decreases in magnitude of both physiological and sevoflurane-induced apoptosis in neonatal mouse S1.

## Introduction

An increasing number of children receive general anesthesia during surgery or examination, and the potential effect of general anesthetics on brain development has aroused widespread concern. Many laboratory studies have demonstrated that general anesthetics can adversely affect the development of the central nervous system in rodents and non-human primates by inducing neuronal apoptosis and affecting synaptic development and plasticity, and even induce permanent cognitive decline (Jevtovic-Todorovic et al., 2003; Kahraman et al., 2008; Brambrink et al., 2012; Raper et al., 2015; Bosnjak et al., 2016; Vutskits and Xie, 2016; Jevtovic-Todorovic, 2018). Clinical studies also suggest that repeated exposure to general anesthesia in infants negatively impacts their learning, cognition, language, and motor ability (McCann et al., 2019; Aksenov et al., 2020). Therefore, it is important to explore the potential influence of general anesthetics on the brain development and determine the possible underlying mechanisms.

Studies have shown that nearly all general anesthetics used in clinical practice have neurotoxic effects on the developing brain and can cause neuronal apoptosis. In the developing cerebral cortex, the vulnerable time window for anesthetic-induced apoptosis coincides with the developmental period of synaptogenesis, which in rodents is the first 2 weeks postnatally (Ikonomidou et al., 1999; Wang et al., 2017; Lee et al., 2021). During the period of synaptogenesis, physiological apoptosis occurs to eliminate excess and inappropriately integrated cells (Southwell et al., 2012; Nikolić et al., 2013). In our previous study, we found that ketamine can increase neuronal apoptosis in the mouse primary somatosensory cortex (S1), and ketamine-induced apoptosis follows the characteristics of physiological apoptosis; the apoptotic neurons were mainly distributed in layer V at postnatal day (P) 5, and the majority were pyramidal neurons, whereas at P9, the apoptosis mainly targeted GABAergic interneurons in layers II-IV (Wang et al., 2017). However, whether other kinds of general anesthetics, in addition to ketamine, also increase apoptosis in neonatal mouse S1 following developmental patterns similar to those of physiological apoptosis, has scarcely been reported.

Sevoflurane is a commonly used inhaled pediatric anesthetic in the clinic. Unlike ketamine which inhibits excitatory glutamate-mediated synaptic transmission through NMDA receptor blockade, sevoflurane mainly exerts modulatory effects on GABA_*A*_ receptors and enhances GABA_*A*_ receptor-mediated inhibitory synaptic transmission (Rudolph and Antkowiak, 2004; Hemmings et al., 2005). Besides, it has been demonstrated that sevoflurane also exerts positive effects on K^+^ channels, such as voltage-gated (Kv) channels and the background/leak or tandem 2-pore (K2P) families (Li et al., 2018). Previous studies in neonatal animal models have indicated that sevoflurane inhalation could induce widespread neuronal apoptosis and even subsequent cognitive impairment (Tao et al., 2014; Wei et al., 2019; Shen et al., 2021). However, there is insufficient information for the comparison of the developmental characteristics between physiological and sevoflurane-induced apoptosis in S1. Similarity in the time course and vulnerable cell populations between physiological and anesthetic-induced apoptosis may suggest common underlying mechanisms. The transcription factor family FoxO (forkhead box, O class), including FoxO1, FoxO3a, FoxO4, and FoxO6, is involved in diverse cellular processes, including apoptosis, cell proliferation, DNA repair, cell cycle, and metabolism (Tzivion et al., 2011). FoxO1 is widely expressed in the developing brain, and impacts neuronal apoptosis, growth, and maturation (Brunet et al., 1999; de la Torre-Ubieta et al., 2010; Xing et al., 2018; Semenza et al., 2021). The activity of FoxO1 is mainly regulated by the PI3K/Akt pathway. PI3K phosphorylates Akt to phosphorylated Akt (p-Akt), and the latter is responsible for inducing phosphorylation of FoxO1, reducing the transcriptional activity of the latter by promoting its subcellular redistribution from the nucleus to the cytoplasm (Hughes et al., 2011; Maiese, 2015; Dong et al., 2018). Activated nuclear FoxO1 controls the expression of numerous downstream genes, which in turn impact multiple cellular processes including apoptosis. PUMA (p53 upregulated modulator of apoptosis) mRNA is among the downstream targets of activated FoxO1 (Hughes et al., 2011). As a Bcl-2 family member, PUMA promotes apoptosis in a p53-dependent and -independent manner in response to a wide variety of stimuli (Yu and Zhang, 2008). However, whether the Akt-FoxO1-PUMA pathway is developmentally involved in regulating both physiological and anesthetic-induced neuronal apoptosis in the neonatal brain is insufficiently documented.

Therefore, in the present study, we investigated the developmental characteristics of sevoflurane-induced apoptosis in S1 of neonatal mouse and explored whether the Akt-FoxO1-PUMA signaling pathway plays a role in regulating the magnitude of both physiological and sevoflurane-induced neuronal apoptosis.

## Materials and Methods

### Experimental Animals

C57BL/6 mice of postnatal days 5 and 9 (P5 and P9, respectively) were used. In a subset of experiments, GAD67-GFP knock-in mice [also known as Gad1^TM 1^.^1*Tama*^; GAD67-GFP (delta neo)], in which enhanced GFP was targeted to the *Gad67* locus that encodes GAD67, were used to investigate the apoptosis of GABAergic neurons (Tamamaki et al., 2003). All the mice were reared on a 12-h light/dark cycle in temperature- and humidity-controlled rooms. To reduce suffering of the animals, we used the minimum number of mice required for statistical accuracy. The procedures adhered to the National Institutes of Health Guide for the Care and Use of Laboratory Animals and were approved by the Ethical Committee for Animal Research of Fudan University.

### Anesthesia

Littermates were randomly assigned to the control and anesthesia groups. The anesthesia group underwent a 4-h exposure to 1.5% sevoflurane in 30% oxygen and 70% room air, and the control group inhaled the carrier gas. Initial experiments did not reveal any sex-based differences in neuroapoptosis, and both female and male littermates were used. A gas analyzer (RGM 5250; Datex-Ohmeda, Louisville, CO, United States) was used to monitor the concentrations of sevoflurane, oxygen, and carbon dioxide. Throughout the experiment, animals were kept in padded acrylic containers equipped with incubators, at a temperature of 37°C.

### Immunohistochemistry, Image Acquisition, and Analysis

Immunohistochemistry and image acquisition were completed with a modification of the procedure described in our previous studies (Wang et al., 2017). Mice pups were deeply anesthetized with 0.7% sodium pentobarbital 6 h after anesthesia initiation and perfused with 0.9% saline followed by 4% paraformaldehyde in phosphate-buffered saline. We subsequently removed the brains, post-fixed them in 4% paraformaldehyde/phosphate buffer solution for 4-6 h at 4°C, and then equilibrated them in 30% sucrose. A cryostat (Leica CM1950, Wetzlar, Germany) was used to cut coronal sections (30 μm) containing the S1. Every 5*^th^* section was immunostained and incubated in a blocking solution containing 5% bovine serum albumin and 0.5% Triton X-100 for 2 h at 37°C. Subsequently, the sections were incubated overnight at 4°C in primary rabbit monoclonal antibody against cleaved caspase-3 (CC3, 9661 L, 1:400; Cell Signaling Technology, Beverly, MA, United States) in 0.3% bovine serum albumin. Sections were washed in phosphate-buffered saline for three times. We then incubated them with Alexa Fluor-conjugated (488 or 568 nm) secondary antibodies at a 1:500 dilution (Thermo Fisher Scientific, Waltham, MA, United States) for 2 h at 37°C. The nuclei were labeled for 30 min at 37°C with TO-PRO-3 (TOPRO, T3605, 1:10,000; Thermo Fisher Scientific).

A Pascal confocal microscope (Zeiss, Jena, Germany) with a 10 × Fluor objective (N.A. = 0.5) or a NiE-A1 plus confocal microscope (Nikon, Tokyo, Japan) with a 10 × Fluor objective (N.A. = 0.45), both with a Z-step of 5 μm, were used to quantify the number of CC3^+^ cells. A Nikon NiE-A1 plus a confocal microscope with a 40 × Plan Fluor oil-immersion objective (N.A. = 1.3) was used at a Z-step of 0.6 μm to estimate the amount of co-localization between CC3^+^ cells and GAD67-GFP. ImageProPlus software (Media-Cybernetics, Rockville, MD, United States) was used to analyze the images by researchers blinded to the experimental conditions. If CC3-stained cells were above the threshold size and displayed a detectable cellular outline, they were considered as CC3^+^. Further, we used TOPRO to measure the image area and identify cortical lamination. To investigate the co-localization of CC3^+^ and GAD67-GFP, all Z-stack layers were checked to ensure colocalization. We imaged and examined approximately 40 CC3^+^ cells per pup. Ten image frames were examined per pup in all investigations. Brain slices from each group were co-processed in all the steps involved in immunostaining, imaging, and data analysis.

### Arterial Blood Gas Measurements

We measured arterial blood gas in the P5 pups at 2- or 4-h following sevoflurane anesthesia. A heparinized 32-gauge hypodermic needle was used to obtain arterial blood from the left ventricle by transcardiac aspiration. A portable clinical analyzer (ABL800FLEX, Radiometer, Copenhagen, Denmark) was used to measure the pH, partial pressure of carbon dioxide, partial pressure of oxygen, oxygen saturation, and HCO_3_^–^ concentration immediately after the arterial blood was obtained.

### Western Blotting

We used intraperitoneal injection of sodium pentobarbital to deeply anesthetize the mice 6 h following sevoflurane or carrier gas inhalation. They were then decapitated, and the regions encompassing the bilateral S1 were removed and stored in liquid nitrogen. We homogenized the tissues in ice-cold radioimmunoprecipitation-assay lysis buffer (Beyotime Biotechnology, Shanghai, China) supplemented with 0.1 mM phenylmethylsulfonyl fluoride protease inhibitors and phosphatase inhibitors. Next, the tissues were lysed on ice for 30 min and centrifuged at 4°C for 10 min to extract total S1 proteins. The samples were then heated at 95°C for 10 min in loading buffer. Subsequently, we filled the 10% sodium dodecyl sulfate-polyacrylamide gel electrophoresis chambers with equal amounts of protein. Samples were separated and transferred to polyvinylidene difluoride membranes by electrophoresis. The membranes were blocked with 5% non-fat milk for 2 h at room temperature and incubated at 4°C with a primary antibody [FoxO1, 1:1000 dilution, CST#2880; Phospho-FoxO1, 1:1000 dilution, CST#84192; Puma (E2P7G), 1:1000 dilution, CST#98672; Akt, 1:1000 dilution, CST#9272; Phospho-Akt (Ser473), 1:1000 dilution, CST#4060] or β-actin (1:500 dilution, Boster#BM3873, Wuhan, China) overnight. The membranes were washed 3 × with tris-buffered saline with Tween 20 and incubated within horseradish peroxidase-conjugated secondary anti-rabbit IgG (1:2000 dilution; Abcam#ab6721, Cambridge, MA, United States) for 2 h at room temperature. The bands were visualized using enhanced chemiluminescence (Thermo Fisher China, Shanghai, China). ImageJ software (National Institutes of Health, Bethesda, MD, United States) was used to perform the densitometry analysis.

### Statistical Analysis

Statistical analyses were performed using Graph Pad Prism 6 (Graph Pad Software, La Jolla, CA, United States). One-way analysis of variance (ANOVA, comparing three or more conditions) followed by Dunnett’s test (all conditions compared to the control) and two-way ANOVA (for comparing two independent variables) were used for assessing statistical significance, followed by Bonferroni’s or Tukey’s multiple comparisons test, as specified in the figure legends. Data are shown as mean ± standard error of the mean. *P* < 0.05 was considered statistically significant, and all the conditions statistically different from controls were reported.

## Results

### Similar Changes in the Magnitude and Laminar Pattern of Sevoflurane-Induced and Physiological Neuronal Apoptosis in S1 During Development

**FIGURE 1:**
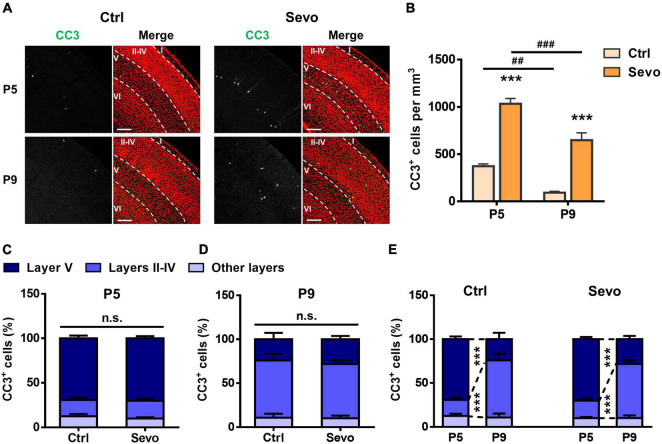
During development, sevoflurane-induced apoptosis follows a lamination pattern similar to that of physiological apoptosis. **(A)** Representative confocal images of coronal primary somatosensory cortex (S1) sections immunolabelled for cleaved caspase-3 (CC3; green) and co-stained for nuclei with TOPRO (red) to visualize cortical lamination. Age and treatment conditions are as indicated. Cortical layers are indicated by Roman numerals, and borders between cortical layers marked using dotted lines. The scale bar is 200 μm. **(B)** Quantitation of the number of CC3^+^ cells per mm^3^ in the S1. Age and treatment conditions are as indicated. *, sevoflurane group compared to control group of the same age; **^#^**, P5 compared to P9 of each treatment condition; ***, *P* < 0.001; **^##^**, *P* < 0.01; **^###^**, *P* < 0.001, using two-way analysis of variance (ANOVA) followed by Bonferroni *post hoc* tests. **(C,D)** Distribution of the proportion of CC3^+^ cells in different cortical layers at P5 **(C)** and P9 **(D).** In each age group, the percentage of CC3^+^ cells in layers II–IV, layer V, or other layers (layer I and layer VI) in the sevoflurane group is not significantly different from that of the control group; *P* > 0.05 by two-way ANOVA followed by Bonferroni *post hoc* tests. **(E)** Replot of the data presented in panels **(C,D)** for the control and sevoflurane groups, showing developmental changes in the apoptosis pattern. ***, *P* < 0.001 by two-way ANOVA followed by Bonferroni *post hoc* tests. Three mice were used per condition, and 10 brain slices from each mouse were quantitated. Data are shown as the mean ± standard error of mean.

Our previous work demonstrated that ketamine-induced neuronal apoptosis in the mouse S1 follows a developmental pattern similar to that of physiological apoptosis, peaking during P5-P7, and shifting from primarily layer V pyramidal neurons at P5 to mostly layers II-IV GABAergic interneurons at P9 (Wang et al., 2017). To explore whether sevoflurane could induce apoptosis in S1 with similar characteristics, P5 and P9 mice were anesthetized by inhalation of 1.5% sevoflurane for 4 h (Sevo), and the control littermates inhaled the carrier gas (30% O_2_ + 70% air) (Ctrl). Cleaved caspase-3 (CC3) plays a central role in the occurrence of apoptosis and is generally considered a marker of apoptosis (Amente et al., 2011). Therefore, we measured the number of CC3^+^ cells in the mouse S1. We found that sevoflurane could significantly increase neuronal apoptosis in S1 of P5 and P9 mice (P5, Ctrl: 373.33 ± 23.18/mm^3^, Sevo: 1033.33 ± 55.43/mm^3^, *P* < 0.001; P9, Ctrl: 93.33 ± 14.51/mm^3^, Sevo: 648.33 ± 77.20/mm^3^, *P* < 0.001; Figures 1A,B) and the extent of apoptosis in both Ctrl and Sevo groups significantly decreased from P5 to P9 (Ctrl, P5: 373.33 ± 23.18/mm^3^, P9: 93.33 ± 14.51/mm^3^, *P* < 0.01; Sevo, P5: 1033.33 ± 55.43/mm^3^, P9: 648.33 ± 77.20/mm^3^, *P* < 0.001; Figures 1A,B).

To further explore whether sevoflurane induced apoptosis following a similar age-dependent laminar pattern as that of physiological apoptosis, the proportion of CC3^+^ cells in different cortical layers were quantitated. The results showed that, in each group, the CC3^+^ cells were mainly distributed in layer V at P5 (Ctrl: 69.34 ± 3.03%, Sevo: 70.19 ± 2.45%, *P* > 0.05; Figures 1A,C), while they were mostly localized in layers II-IV at P9 (Ctrl: 65.08 ± 7.53%, Sevo: 61.58 ± 4.30%, *P* > 0.05; Figures 1A,D). The proportion of CC3^+^ apoptotic cells in different S1 layers was plotted from P5 to P9 to better compare the developmental changes in the laminar pattern of apoptosis. Regardless of anesthetization, a significant shift of CC3^+^ cells from layer V to layers II-IV was noted going from P5 to P9 (Figure 1E). For both ages and conditions, CC3^+^ cells in layers I and VI accounted for only a small proportion, and the percentage did not change with age or treatment condition (Figure 1).

To determine whether sevoflurane-induced neuronal apoptosis was caused by hypoxia or hypercapnia, arterial blood gas analysis was performed in mice 2 and 4 h following sevoflurane anesthesia. The results showed that 1.5% sevoflurane anesthesia did not affect levels of pH, pressure of carbon dioxide (PaCO_2_), partial pressure of oxygen (PaO_2_), HCO_3_^–^, or oxygen saturation (SaO_2_) in P5 mice (Table 1). The results indicated that the mouse pups could maintain normal respiration and oxygenation during anesthesia and ruled out the influence of hypoxia or hypercapnia on neuronal apoptosis.

**TABLE 1:** Arterial blood-gas analysis of postnatal-day-5 (P5) mice following 1.5% sevoflurane.

Parameter	Ctrl		Sevo	
	2 h	4 h	2 h	4 h
pH	7.53 ± 0.02	7.60 ± 0.02	7.49 ± 0.05	7.47 ± 0.03
PaCO_2_ (mmHg)	26.58 ± 3.98	22.48 ± 2.48	36.38 ± 4.32	32.50 ± 4.47
PaO_2_ (mmHg)	109.50 ± 23.99	127.25 ± 20.13	100.25 ± 16.96	115.00 ± 15.32
HCO_3_^–^ (mM)	23.75 ± 0.66	22.63 ± 0.81	22.83 ± 1.01	22.28 ± 1.58
SaO_2_ (%)	97.25 ± 1.56	99.00 ± 0.47	96.43 ± 1.95	97.40 ± 1.60

*The parameters measured and conditions are as indicated. Measurements were taken at 2 h and 4 h following anesthesia. Four to ten mice were used per condition. No significant differences were detected between the control and anesthesia conditions for any parameter. P > 0.05, one-way analysis of variance (ANOVA), followed by Dunnett’s multiple comparisons test.*

### The Vulnerability of GABAergic Neurons to Apoptosis Changes During Development

**FIGURE 2:**
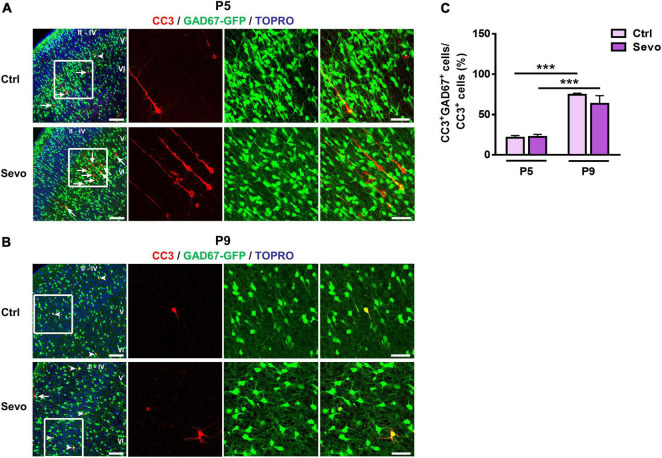
Vulnerability of GABAergic neurons to sevoflurane-induced apoptosis is age-dependent. **(A,B)** Representative confocal images of S1 sections labeled for cleaved caspase-3 (CC3, red), GAD67-GFP (green), and TOPRO (blue) at P5 **(A)** and P9 **(B)**. The cortical layers are indicated by Roman numerals. Conditions are as indicated. White arrowheads indicate CC3^+^ cells co-localized with GAD67-GFP, while the white arrows indicate those that are not co-localized. The scale bar is 100 μm. Zoomed images of boxed regions are presented to the right of each panel with scale bars = 50 μm. **(C)** Proportion of CC3^+^ cells co-labeled with GAD67-GFP in all cortical layers. Three to four mice were used for each condition. ***, *P* < 0.001, using two-way analysis of variance followed by Bonferroni *post hoc* tests. Data are shown as the mean ± standard error of the mean.

The above results showed that physiological and sevoflurane-induced apoptosis in S1 exhibited similar age-dependent changes in magnitude and laminar pattern. Next, we explored whether the cell-type vulnerability to sevoflurane-induced apoptosis also changed developmentally as observed in physiological apoptosis. GAD67-GFP transgenic mouse littermates inhaled sevoflurane (1.5%, 4 h) and carrier gas (30% O_2_ + 70% air), respectively. The results showed that at P5, GAD67-GFP^+^ interneurons accounted for less than one-third of the CC3^+^ apoptotic neurons in S1 (Ctrl: 21.25% ± 2.65%, Sevo: 22.27% ± 3.15%; Figures 2A,C), and there was no significant difference between the control and sevoflurane groups (*P* > 0.05; Figures 2A,C). Most of the apoptotic neurons had typical pyramidal neuron morphology, with triangular cell bodies and long apical dendrites (Figure 2A). However, by P9, the majority of the apoptotic neurons were GAD67-GFP^+^ interneurons in both control and sevoflurane groups (Ctrl: 74.54 ± 1.81%, Sevo: 73.32 ± 1.61%, *P* > 0.05; Figures 2B,C).

These results indicated that the cell type susceptible to both physiological and sevoflurane-induced apoptosis in S1 shifted from mostly pyramidal neurons at P5 to GABAergic interneurons at P9. Sevoflurane augmented neuronal apoptosis in S1 without affecting the developmental switch in the cell-type specificity of physiological apoptosis.

### The Akt-FoxO1-PUMA Pathway Is Developmentally Affected by Sevoflurane

**FIGURE 3:**
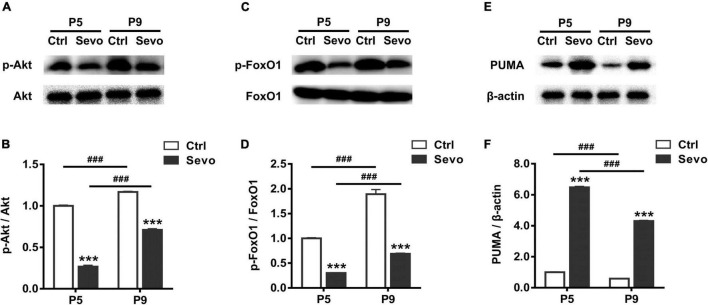
Developmental changes in the Akt–FoxO1–PUMA pathway in regulating physiological and sevoflurane-induced apoptosis. Immunoblots and quantitation of phosphorylated Akt **(A,B)**, phosphorylated FoxO1 **(C,D)**, and PUMA **(E,F)** from the S1 region of control and sevoflurane-anesthetized mice at P5 and P9, respectively. *, sevoflurane group compared to control group of the same age; **^#^**, P5 compared to P9 in each treatment condition; ***, *P* < 0.001; **^###^**, *P* < 0.001, using two-way analysis of variance followed by Tukey’s multiple comparisons test. Four mice were used per condition. Data are shown as the mean ± standard error of the mean.

The developmental similarity of sevoflurane-induced apoptosis and physiological apoptosis in time-course, laminar pattern, and cell-type specificity implied the possibility of common proapoptotic mechanisms. The PI3K/Akt-FoxO1 signaling pathway plays an essential role in regulating cell apoptosis by modulating the expression of the downstream target PUMA (Hughes et al., 2011). Akt, phosphorylated by PI3K (p-Akt), inactivates FoxO1 by inducing its phosphorylation, leading to low expression of the proapoptotic factor PUMA. Since sevoflurane significantly increased the number of apoptotic cells in S1 at both P5 and P9, and the magnitude of either physiological or sevoflurane-induced apoptosis significantly decreased at P9 compared to P5 (Figures 1A,B), we next explored whether the Akt-FoxO1-PUMA pathway participated in sevoflurane-induced apoptosis or the developmental reduction of apoptosis. We showed that, compared with control littermates, in the S1 of sevoflurane-anesthetized mice, the levels of both p-Akt and phosphorylated FoxO1 (p-FoxO1) significantly decreased at P5 (Ctrl vs Sevo: p-Akt, 1.00 ± 0.01 vs 0.27 ± 0.02, *P* < 0.001; p-FoxO1, 1.00 ± 0.01 vs 0.30 ± 0.01, *P* < 0.001) and P9 (Ctrl vs Sevo: p-Akt, 1.17 ± 0.01 vs 0.71 ± 0.01, *P* < 0.001; p-FoxO1, 1.89 ± 0.09 vs 0.69 ± 0.01, *P* < 0.001) (Figures 3A–D). Accordingly, the level of the proapoptotic downstream product PUMA was significantly greater in sevoflurane-anesthetized mice than in control littermates at both P5 (Ctrl vs Sevo, 1.00 ± 0.02 vs 6.48 ± 0.06, *P* < 0.001) and P9 (Ctrl vs Sevo, 0.59 ± 0.02 vs 4.30 ± 0.05, *P* < 0.001) (Figures 3E,F). The reduction of p-Akt and p-FoxO1, along with an increase in the PUMA levels, suggests that the Akt-FoxO1-PUMA pathway might mediate sevoflurane-induced apoptosis in S1 of P5 and P9 pups. To determine whether this pathway is also involved in regulating the reduction of either physiological or sevoflurane-induced apoptosis from P5 to P9, we subsequently assayed the developmental changes in levels of p-Akt, p-FoxO1, and PUMA. We showed that in both control and sevoflurane groups, the level of p-Akt (P5 vs P9: Ctrl, 1.00 ± 0.01 vs 1.17 ± 0.01, *P* < 0.001; Sevo, 0.27 ± 0.02 vs 0.71 ± 0.01, *P* < 0.001) and p-FoxO1 (P5 vs P9: Ctrl, 1.00 ± 0.01 vs 1.89 ± 0.09, *P* < 0.001; Sevo, 0.30 ± 0.01 vs 0.69 ± 0.01, *P* < 0.001) in the S1 was significantly higher at P9 than at P5 (Figures 3A–D), whereas the level of PUMA significantly declined from P5 to P9 (P5 vs P9: Ctrl, 1.00 ± 0.02 vs 0.59 ± 0.02, *P* < 0.001; Sevo, 6.48 ± 0.06 vs 4.30 ± 0.05, *P* < 0.001) (Figures 3E,F). These results indicate that the Akt–FoxO1–PUMA signaling pathway may play a role in the developmental reduction of both physiological and sevoflurane-induced apoptosis.

## Discussion

It is well documented that nearly all commonly used general anesthetics are able to cause widespread apoptotic neurodegeneration in various newborn animal species (Jevtovic-Todorovic et al., 2013; Lin et al., 2014; Lee et al., 2017). The window of vulnerability to apoptosis induced by general anesthetics coincides with the brain growth spurt period, which occurs during the first 2 postnatal weeks in rodents (Dobbing and Sands, 1979). Physiological apoptosis also occurs during this period, and is essential for the establishment of the normal central nervous system structure (Kuida et al., 1996). The hypernomic neuronal apoptosis induced by general anesthetics may disrupt brain development and subsequently cause neurocognitive impairment (Creeley, 2016).

Since physiological apoptosis also occurs during the period when brain was vulnerable to anesthetic-induced apoptosis, it is important to explore whether anesthetic-induced apoptosis targets the same or different populations in comparison with those targeted by physiological apoptosis. Our previous work compared the characteristics of ketamine-induced apoptosis in the mouse S1 with those of physiological apoptosis (Wang et al., 2017). We showed that ketamine-induced and physiological apoptosis shared similar developmental patterns, peaking during P5–P7, becoming insignificant by P12, and shifting from primarily layer V pyramidal neurons at P5 to mostly layers II-IV GABAergic interneurons by P9 (Wang et al., 2017). Ketamine mediates its effects mainly by blocking N-methyl-D-aspartate receptors (Ikonomidou et al., 1999), and we wondered whether anesthetic agents acting primarily on GABA_*A*_ receptors have similar characteristics regarding the induction of apoptosis in neonatal mouse S1. Thus, herein, the developmental characteristics of sevoflurane-induced apoptosis in the S1 were explored and compared with those of physiological apoptosis. We found that a 4-h 1.5% sevoflurane anesthesia significantly augmented neuronal apoptosis in the S1, and, consistent with the developmental changes in magnitude of physiological apoptosis, sevoflurane-induced apoptosis was more prominent at P5 than at P9 (Figure 1). Moreover, the cell types vulnerable to sevoflurane-induced apoptosis at P5 included mainly pyramidal neurons in layer V, whereas at P9, mainly GABAergic interneurons in layers II-IV were involved (Figures 1, 2). Although sevoflurane and ketamine act mainly on different targets, they induce apoptosis in the neonatal mouse S1 similarly to physiological apoptosis in terms of developmental time-course, lamination, and cell-type specificity (Wang et al., 2017), which is the major innovation point of the current study.

Referring to sevoflurane-induced neuronal apoptosis, an immediately arising question is whether isoflurane has comparable effects as sevoflurane on inducing neuronal apoptosis during postnatal development. Isoflurane is also a commonly used volatile anesthetic in clinical and has frequently been reported to induce widespread apoptotic neurodegeneration when administered to neonatal animals. Despite the similar effects on enhancing GABA_*A*_ receptor function and activating certain types of K^+^ channels, isoflurane has been reported to induced a significantly greater amount of apoptotic neurons in different brain regions than an equipotent exposure of sevoflurane (Liang et al., 2010; Zhao et al., 2020). Isoflurane induced neuronal apoptosis in neonatal brain mainly by activation the mitochondrial pathway, while sevoflurane is more prone to causing endoplasmic reticulum (ER) stress-induced apoptosis (Zhao et al., 2020). In terms of the vulnerable cell population targeted by isoflurane, more detailed observation have been reported on neonatal non-human primates (NHPs). Exposure of P6 rhesus macaques to isoflurane significantly increased apoptosis of neurons and oligodendrocyte (Brambrink et al., 2010; Noguchi et al., 2017). Oligodendrocyte apoptosis was uniformly distributed throughout the white matter whereas neuroapoptosis occurred mainly in the cortex, caudate, putamen and thalamus (Noguchi et al., 2017). In the temporal cortex of the isoflurane-exposed rhesus macaques brain the laminar pattern and cell-type specificity are also evident, with GABAergic interneurons in layer II and pyramidal neurons in layer IV being the most severely affected (Brambrink et al., 2010). However, whether and how the laminar pattern and cell-type specificity of isoflurane-induced apoptosis changed during development are waiting for exploration. In addition to general anesthetics, ethanol has also been involved in inducing widespread neuronal apoptosis when exposed to neonates in age- and dose-dependent manners (Olney et al., 2002a,b; Lebedeva et al., 2017; Britton and Miller, 2018). Previous studies investigating the age-dependent apoptosis induced by ethanol in the cerebral cortex reported switch in laminar pattern from layer V at P4-P7 to layer II/III at P10-P13, which is similar with our observation on ketamine- and sevoflurane-induced apoptosis (Lebedeva et al., 2017; Britton and Miller, 2018). However, these studies did not assay whether the apoptotic neurons were pyramidal or GABAergic.

Previous studies exploring developmental changes in cell numbers in the cerebral cortex reported most significant changes in cortical layers II-IV during P5-P10, but they did not assay whether the apoptotic layer II-V neurons were pyramidal or GABAergic, nor did they quantify whether the number of apoptotic cells in different layers changed during development (Ferrer et al., 1992; Ikonomidou et al., 1999). Consistent with our observation, Blanquie et al. showed that the developmental apoptosis of cortical neurons peaked at P5-P6, most of which were GAD67-negative, glutamatergic neurons in layer V of S1 (Blanquie et al., 2017b). However, they did not show whether the cell-type specificity changed with development. Wong et al. reported that most pyramidal neurons undergo apoptosis between P2 and P5 in the mouse cortex, while the period of apoptotic neurodegeneration for cortical interneurons is slightly later compared to pyramidal neurons, with most apoptosis occurring between P5 and P10 (Wong et al., 2018).

In our previous and the current studies, a shift in the lamination pattern of apoptotic cells from mostly layer V pyramidal neurons at P5 to mostly layers II-IV GABAergic interneurons at P9 could be observed in physiological, ketamine- and sevoflurane-induced apoptosis in S1. The similarity does not negate potential adverse effects of anesthesia-induced apoptosis on neural circuit formation and function, however, it may suggest potentially similar underlying mechanisms. What physiological mechanism or process could underlie this developmental change? Since neurons migrated into the cortical plate in an inside-out manner that earlier generated neurons occupy the deeper layers and later generated neurons migrate to form the upper layers of the cortex, neurons in the upper layers are generally less mature than those in the deeper layers (Kriegstein and Noctor, 2004; Wonders and Anderson, 2006). Consistent with the inside-out developmental pattern, it has been shown that the earlier born medial ganglionic eminence (MGE)-derived GABAergic neurons mostly populate deep layers (V-VI) whereas later born MGE cells mostly populate superficial layers (II-IV) (Bartolini et al., 2013). The apoptosis pattern we observed is consistent with developmental trajectory, in that deep layer neurons are more vulnerable at earlier ages. In terms of the developmental changes in cell-type specificity of apoptosis, recent experiments showed that, rather than extrinsic competition-based mechanisms, GABAergic interneurons went through apoptosis drived by an intrinsic timer when they reach a specific maturation stage (Song et al., 2012; Southwell et al., 2012). The cell age 11-18 days interneurons are more vulnerable to apoptosis (Southwell et al., 2012). We surmise that the similar intrinsic program may also exist to drive the apoptosis of pyramidal neurons. Then, why general anesthetics can increase neuronal apoptosis following similar laminar pattern and cell-type specificity?

During the early postnatal period, neural activity, both in the form of spontaneous electrical activity and sensory stimulation, is critical to neuronal survival (Blanquie et al., 2017a). Inhibition of neural activity mainly through blocking NMDA receptor and/or enhancing GABA_*A*_ receptor functions plays a critical role in general anesthetic-induced neuronal apoptosis (Jevtovic-Todorovic et al., 2013). However, this could not be sufficient to explain the age-dependent changes in laminar pattern and cell-type specificity of ketamine- and sevoflurane-induced apoptosis in S1. Previous study investigated the effect of isoflurane on late- and adult-generated neurons in dentate gyrus (DG) and olfactory bulb of newborn, juvenile, and adult mice, and found vulnerability to isoflurane-induced apoptosis was cell age-specific, rather than organism age-specific (Hofacer et al., 2013). Neurons with cellular age 13-15 days were most vulnerable to anesthetic-induced apoptosis, although the cell types were not distinguished (Hofacer et al., 2013). Interestingly, the most vulnerable cell ages exactly overlaps between anesthetic-induced neuronal apoptosis and physiological apoptosis of GABAergic neurons. We surmise that an intrinsic program may regulate neuronal apoptosis during the rapid synaptogenesis of immature brains, and anesthetics (especially ketamine and sevoflurane) may augment the developmental apoptosis by lowering the threshold of the intrinsic program of apoptosis.

The other innovation point of our study is that we found the Akt-FoxO1-PUMA signaling pathway may participate in mediating sevoflurane-induced apoptosis. The PI3K-Akt signaling pathway is extremely important in mediating survival signals in multiple cell types, including neurons (Brunet et al., 2001). P-Akt exerts an anti-apoptotic effect by phosphorylating numerous downstream targets. Previous studies reported that the inactivation of the PI3K-Akt-GSK3β and PI3K-Akt-mTOR pathways were involved in general-anesthetic-induced neurotoxicity (Xing et al., 2020; Zhang et al., 2021; Zhu et al., 2021). Our results add to the existing knowledge by showing that the Akt-FoxO1-PUMA pathway participates in mediating sevoflurane-induced neuronal apoptosis in S1 of neonatal mice. FoxO transcription proteins play crucial roles in a broad range of cellular functions (Accili and Arden, 2004), and are considered important factors regulating memory formation and cognitive functions (Manolopoulos et al., 2010; Fang et al., 2019). FoxO1, a representative member with abundant expression, has been demonstrated to be involved in apoptosis induction, reactive oxygen species suppression, neural progenitor cell proliferation, and more (Santo and Paik, 2018; Semenza et al., 2021). The PI3K/Akt pathway is one of the prime regulators of FoxO1 activity. P-Akt by PI3K subsequently phosphorylates FoxO1, resulting in its nuclear exclusion and cytoplasmic sequestration. Thus, an increase in p-FoxO1 adversely regulates the expression of target genes (Brunet et al., 1999). PUMA is one of the numerous FoxO1 downstream targets whose expression is related to triggering apoptosis by activating the proapoptotic family member Bax (Hughes et al., 2011; Kon et al., 2019). Accordingly, we demonstrated that the level of p-Akt and its immediately downstream target p-FoxO1 was significantly attenuated following sevoflurane anesthesia, which is responsible for the striking elevation of PUMA expression we observed in S1 at P5 and P9 (Figure 3). The increase in PUMA levels may contribute to the greater number of CC3^+^ apoptotic neurons observed in S1 of sevoflurane-anesthetized mouse pups. Moreover, our results demonstrated that the Akt-FoxO1-PUMA pathway could also be involved in mediating the developmental reduction of both physiological and sevoflurane-induced apoptosis in the S1. Supporting the fact that CC3^+^ apoptotic neurons significantly decreased from P5 to P9 in both control and sevoflurane-anesthetized pups (Figure 1), we observed a significant increase in p-Akt and p-FoxO1 levels, and consequently, a notable attenuation in the downstream PUMA levels in S1 of both groups from P5 to P9 (Figure 3). The developmental reduction in levels of the proapoptotic PUMA is assumed to be responsible for the age-dependent decrease of both physiological and sevoflurane-induced apoptosis. Together, these results provide evidence for the roles of the Akt-FoxO1-PUMA signaling pathway in mediating sevoflurane-induced apoptosis, as well as the reduction of physiological and sevoflurane-induced apoptosis in S1 following the postnatal development.

Some limitations still exist in our study: Firstly, we used a relatively lower concentration of sevoflurane (1.5%) to minimize the adverse effects on cardiopulmonary physiology and mortality. This may underestimate the effect of sevoflurane on neurodegeneration. Moreover, despite the developmental changes in lamination pattern of ketamine-induced apoptosis in S1 are not affected by drug concentrations, whether that would be the case in sevoflurane-induced apoptosis still needs to explore. Secondly, it has been well-documented that PUMA can mediate cell death in an p53-dependent and -independent manner (Yu and Zhang, 2008), and consistent with the increase in neuronal apoptosis induced by sevoflurane, we observed the significantly higer levels of PUMA expression in S1. However, the direct evidence of PUMA takes part in mediating sevoflurane-induced apoptosis is lacking. Thirdly, as discussed above, the laminar pattern and cell-type specificity of both physiological and sevoflurane-induced apoptosis may mediated by similar intrinsic programs, which should be deeply explored by further *in vivo* and *in vitro* experiments.

In conclusion, our results demonstrate that sevoflurane-induced neuronal apoptosis in the neonatal mouse S1 follows patterns similar to those of physiological apoptosis in terms of the time-course, lamination, and cell-type specificity. Moreover, the Akt-FoxO1-PUMA signaling pathway probably participates in mediating apoptosis induced by sevoflurane and contributes to the developmental reduction of both physiological and sevoflurane-induced apoptosis in S1.

## Data Availability Statement

The original contributions presented in the study are included in the article/supplementary material, further inquiries can be directed to the corresponding author.

## Ethics Statement

The animal study was reviewed and approved by the Ethical Committee for Animal Research of Fudan University.

## Author Contributions

QW and YW contributed to conception and design of the study. QW and YL organized the database. QW, YL, and HT performed the statistical analysis. QW, YL, and YW wrote the manuscript. All authors contributed to manuscript revision, read, and approved the submitted version.

## Conflict of Interest

The authors declare that the research was conducted in the absence of any commercial or financial relationships that could be construed as a potential conflict of interest.

## Publisher’s Note

All claims expressed in this article are solely those of the authors and do not necessarily represent those of their affiliated organizations, or those of the publisher, the editors and the reviewers. Any product that may be evaluated in this article, or claim that may be made by its manufacturer, is not guaranteed or endorsed by the publisher.
